# The Implementation of Birdsong: A Non-Pharmacological Intervention for Persons With Dementia

**DOI:** 10.1093/geroni/igaa057.2424

**Published:** 2020-12-16

**Authors:** Catherine Tompkins, Joan Thomas, Christina Cole, Mei Qiu

**Affiliations:** 1 George Mason University, Fairfax, Virginia, United States; 2 Birmingham Green, Manassas, Virginia, United States

## Abstract

Birdsong is a person-centered, nonpharmacological intervention for people with dementia. Using a touch-screen tablet, residents with dementia are stimulated with content customized based on individual interests such as sports, videos, music or brain games. The increased stimulation is expected to redirect negative behaviors, decrease the use of anti-psychotic medications and increase positive emotions. Birdsong is being implemented in a 100% Medicaid funded, long-term care facility. Two 10-week experimental design studies were implemented with 39 residents. Preliminary findings suggest a lack of independence with the tablets among the residents, but positive stimulation of emotions and redirection of negative behaviors with assistance of staff or volunteers. A team of practitioners, faculty and student researchers continue to examine the intervention, its implementation and effects on this population by examining the Minimum Data Set (MDS data) and observations of the residents as they utilize Birdsong.

